# Cost-effectiveness of novel vaccines for tuberculosis control: a decision analysis study

**DOI:** 10.1186/1471-2458-11-55

**Published:** 2011-01-26

**Authors:** Chia-Lin Tseng, Olivia Oxlade, Dick Menzies, Anne Aspler, Kevin Schwartzman

**Affiliations:** 1 Respiratory Epidemiology and Clinical Research Unit, Montreal Chest Institute, Montreal, Canada; 2 Faculty of Medicine, McGill University, Montreal, QC, Canada; 3 Department of Epidemiology, Biostatistics, and Occupational Health, McGill University, Montreal, QC, Canada; 4 Respiratory Division, McGill University, Montreal, QC, Canada; 5 Internal Medicine Residency Training Program, Faculty of Medicine, University of Toronto, Toronto, ON, Canada

## Abstract

**Background:**

The development of a successful new tuberculosis (TB) vaccine would circumvent many limitations of current diagnostic and treatment practices. However, vaccine development is complex and costly. We aimed to assess the potential cost effectiveness of novel vaccines for TB control in a sub-Saharan African country - Zambia - relative to the existing strategy of directly observed treatment, short course (DOTS) and current level of bacille Calmette-Guérin (BCG) vaccination coverage.

**Methods:**

We conducted a decision analysis model-based simulation from the societal perspective, with a 3% discount rate and all costs expressed in 2007 US dollars. Health outcomes and costs were projected over a 30-year period, for persons born in Zambia (population 11,478,000 in 2005) in year 1. Initial development costs for single vaccination and prime-boost strategies were prorated to the Zambian share (0.398%) of global BCG vaccine coverage for newborns. Main outcome measures were TB-related morbidity, mortality, and costs over a range of potential scenarios for vaccine efficacy.

**Results:**

Relative to the status quo strategy, a BCG replacement vaccine administered at birth, with 70% efficacy in preventing rapid progression to TB disease after initial infection, is estimated to avert 932 TB cases and 422 TB-related deaths (prevention of 199 cases/100,000 vaccinated, and 90 deaths/100,000 vaccinated). This would result in estimated net savings of $3.6 million over 30 years for 468,073 Zambians born in year 1 of the simulation. The addition of a booster at age 10 results in estimated savings of $5.6 million compared to the status quo, averting 1,863 TB cases and 1,011 TB-related deaths (prevention of 398 cases/100,000 vaccinated, and of 216 deaths/100,000 vaccinated). With vaccination at birth alone, net savings would be realized within 1 year, whereas the prime-boost strategy would require an additional 5 years to realize savings, reflecting a greater initial development cost.

**Conclusions:**

Investment in an improved TB vaccine is predicted to result in considerable cost savings, as well as a reduction in TB morbidity and TB-related mortality, when added to existing control strategies. For a vaccine with waning efficacy, a prime-boost strategy is more cost-effective in the long term.

## Background

Nearly a third of the world's population harbors the *Mycobacterium tuberculosis *bacillus, and about 1.7 million people die of tuberculosis (TB) each year [1]. Since the early 1990s, inconsistent treatment and the consequent emergence of drug resistance, and widespread HIV infection have contributed to the global epidemic. Other major challenges include limited diagnostic tools, and suboptimal provider/patient adherence with recommended diagnostic and treatment interventions [2]. The World Health Organization's recommended strategy of directly observed treatment, short course (DOTS) has reduced incidence in most world regions; however, TB control in sub-Saharan Africa and in the former Soviet Union continues to be a particular concern [3,4]. In response to these challenges, the World Health Organization (WHO) has broadened its approach to TB control, for example by considering HIV-TB co-infection, smear-negative TB and treatment for latent infection [5]. In addition, there is intensive research into novel diagnostic, therapeutic, and preventive interventions. The development of an effective TB vaccine holds great appeal; by preventing TB infection and/or disease, it would circumvent limitations of current diagnostic and treatment strategies.

The only currently licensed TB vaccine, bacille Calmette-Guérin (BCG), has proven at least modestly efficacious in preventing tuberculous meningitis and disseminated disease in young children, although estimates of protection vary [6,7]. It has very limited efficacy in preventing adult pulmonary disease, the form of disease that is contagious, and hence fuels the continuing epidemic. Based on current development timelines, next-generation TB vaccines may become available in the next 5-7 years [8,9]. Because a suboptimal vaccine is already in widespread use, an improved vaccine is likely to attain the same or even better population coverage. However, costs for development, testing, and implementation may be substantial. Given the limited resources available for TB control, it is relevant to consider likely costs and public health benefits of a novel vaccine, based on currently available information. If vaccine development is likely to prove very expensive, with public health benefits accruing only in the distant future, then other interventions may be accorded higher priority.

Using a simulation model, we aimed to predict health outcomes and costs with different scenarios for the introduction of a novel TB vaccine in a sub-Saharan African country. We were particularly interested in the impact of varying scenarios for vaccine efficacy, and for the mechanism and timing of vaccine effect. As a case study, we estimated TB-related morbidity, mortality, and costs of a novel TB vaccine introduced in Zambia, a country with high HIV prevalence (9.4%) and very high annual TB incidence (247 smear positive cases/100,000) [1,10]. We projected changes in TB morbidity, mortality, and cost when an improved vaccine replaces the current BCG vaccination program, and supplements the current DOTS program, over a range of scenarios for vaccine efficacy.

## Methods

### General description of the model

We developed a decision analysis model, incorporating multiple Markov processes, using TreeAge Pro Suite 2007 (TreeAge Software, Williamstown, MA). The model estimated the probability of developing active TB disease, TB mortality, and associated costs over a period of 30 years for the existing TB control strategy (BCG for newborns, plus DOTS based on smear diagnosis and standard drug regimens), and for two alternative country wide vaccination strategies. We simulated a hypothetical fixed cohort of newborns who joined the existing population of Zambia. The characteristics of the Zambian population used for this simulation are summarized in Table 1. The analysis was conducted from a societal perspective, including both direct and indirect costs. All future expenditures and outcomes were discounted at a rate of 3% annually [11].

**Table 1:** Epidemiologic and Program Data for Zambia

Variable	Value	Source
Population (2005)	11,478,000	[56]
Live births/1,000 population (2007)	40.78	[57]

Live births per year	468,073	Calculated from [56,57]

Percent of global BCG vaccine coverage	0.398%	[34]

Percent of target population vaccinated with BCG	92%	[34]

Gross National Income per capita (US$) 2005	$500	[58]

Life expectancy at birth (years)	38.4	[58]

All cause mortality	Age specific	[59]

New estimated TB smear-positive incidence per 100,000 (2005)	247	[1]

Annual risk of TB infection (ARI)	4.94%	Calculated from [1,16]

Probability of being diagnosed and treated for LTBI	1%	[20]*

Completion of LTBI treatment	67%	[21]

HIV prevalence (2006)	9.4%	[10]

HIV incidence per annum	0.96%	Calculated from [22]

DOTS coverage (2005)	100%	[1]

DOTS case detection rate (2005)	52%	[1]

Initial drug resistance		
Single drug resistance	8.5%	[60]
Multi drug resistance†	1.8%	[61]

DOTS new case treatment outcomes (2005)		[1]
Cure/complete	83%	
Default/transfer/not evaluated	8%	
Die	8%	
Fail	1%	

DOTS re-treatment outcomes (2005)		[1]
Cure/complete	78%	
Default/transfer/not evaluated	7%	
Die	13%	
Fail	2%	

*This assumes only dually infected individuals (HIV and TB) whose HIV infection is detected and are tuberculin tested will be treated (based on a Haitian study)

†Defined as resistance to isoniazid and rifampin, with or without other drug resistance

For the purpose of the primary analysis, current epidemiologic and tuberculosis control parameters (Table 1) were assumed to remain constant over the simulation period: annual risk of TB infection, DOTS coverage, case detection rate, treatment outcomes, smear-positive incidence, HIV seroprevalence, and prevalence of initial TB drug resistance. The status quo included the protective effects of existing BCG vaccination, at the current level of vaccine coverage and with a presumed 50% reduction in the risk of primary progression to pulmonary disease, tuberculous meningitis or disseminated disease during early childhood [12,13]. The reduction in risk conferred by existing BCG vaccination is assumed to apply for a total duration of 10 years, but its protective effect is assumed to wane linearly to zero over this period of time.

In primary analysis, the vaccination strategies that we compared with the existing TB control program were, 1) vaccination with a novel TB vaccine at birth, and 2) vaccination with a novel vaccine at birth plus a booster dose at age 10. Both comparison strategies were assumed to cover the same proportion of the target population as the current BCG program. The protective effect of all vaccine doses was assumed to be immediate, and would then wane linearly to zero over a period of 10 years. In the primary analysis, the replacement TB vaccine for neonates was assumed to have an initial efficacy of 70% for preventing rapid progression to TB disease. Hence the replacement vaccine would work similarly to BCG, but with greater efficacy. As a result, the incidence of contagious smear-positive pulmonary disease will remain largely constant during the first years after introduction of the novel vaccine. This is because young children develop primary, disseminated and/or meningeal forms of the disease which are rarely contagious.

The booster dose administered at the age of 10 was also assumed to be 70% efficacious, and to work by preventing rapid progression to active disease. Hence it would only be of benefit to persons who had not yet acquired latent TB infection by age 10.

Other potential mechanisms for vaccine action could be to reduce: 1) acquisition of initial infection following exposure to *Mycobacterium *tuberculosis; or 2) late reactivation of longstanding latent TB infection. These alternate mechanisms of vaccine action, and their impact on health outcomes and costs, were considered in sensitivity analyses. For this reason, the time frame of all analyses was 30 years, so that potential benefits related to protection against acquiring TB infection and to prevention of late reactivation could be tracked into early adulthood. However, for the primary analysis we also compared outcomes at 5, 10, and 20 years.

Preliminary data suggest some novel TB vaccines currently under development will be safer than BCG for administration to HIV-positive individuals [8,9]. The model assumed all vaccines to have similar protective efficacy in individuals with early HIV infection to that in seronegative persons. We assumed that the vaccine had no effect on the development of active TB in persons with clinical AIDS, based on severe impairment of cell-mediated immunity. To the extent that this could underestimate vaccine benefit, this was a conservative assumption.

### Model health states, transitions, and calculations

In the model, TB-related health states, along with key pathogenetic assumptions corresponding to Markov state transition probabilities (e.g. risk of acquiring tuberculosis infection, subsequent risk of active TB) were as previously described [14,15], modified and extended to include vaccination (Table 2).

**Table 2:** Model Pathogenetic Variables and Assumptions

Variable	Value	Source
**HIV Infection**		
Annual risk of progression - asymptomatic to AIDS	7%	[22,62]
Annual risk of death - early HIV (asymptomatic)	4.6%	[22]
Annual risk of death - clinical AIDS	22%	[22]
Median survival with early HIV	9.8 yrs	[22,62]
Median survival with clinical AIDS	9 months	[22]

**Risk of developing active TB disease**		
**HIV uninfected**		
Within 2 years of new TB infection	5%	[17,63]
Within 2 years of re-infection after cured TB disease	1%	[64,65]
Late re-activation from longstanding latent TB*	0.1%/year	[18,19]

**Early HIV**		
Within 2 years of new TB infection	33%	Extrapolated
Within 2 years of re-infection after cured TB disease	33%	Assumption
Late re-activation from longstanding latent TB*	3.4%/year	[23,66,67]

**Clinical AIDS**		
Within 2 years of new TB infection	100%	[25,68-71]
Within 2 years of re-infection after cured TB disease	100%	Assumption
Late re-activation from longstanding latent TB*	33%/year	[23]

**Untreated Smear Positive TB Outcomes (HIV-negative)**		
Spontaneous resolution	25%	[72]
Relapse after spontaneous resolution	2.5%/year	[72,73]
Mortality rate within 2 years	33% at 1 year; 50% at 2 years	[74]

**Untreated Smear Positive TB Outcomes (HIV-positive)**		
Spontaneous resolution	0%	Assumption
Mortality rate within 2 years	100%	Assumption

**Treated Smear Positive TB Outcomes (HIV-negative)**		
Relapse after cure (total over next 2 years)	3.0%	[75-79]
Cure rate if default (single drug resistant or drug sensitive)**	62.4%	[80-83]

**Effect of Drug Sensitivity on Treatment Outcomes**		
Relative risk of treatment failure - single drug resistant	2.0	[84]
Relative risk of treatment failure - multi-drug resistant	10.5	[84]
Relative risk of death - single drug resistant	1.0	[84]
Relative risk of death - multi-drug resistant	4.5	[84]

**Multi-Drug Resistant TB Treatment Outcomes**		
Completed/Cured	68.6%	[85]
Default/Failed/Transferred	17.1%	[85]
Died	14.2%	[85]

**Treated Smear Positive TB Outcomes (HIV-positive)**		
Relative risk of death during TB treatment with HIV infection	2.25	[24,26,86,87]
Relapse after successful TB treatment (cured)	3.1%	[88-90]

* Assumes rate of reactivation beyond 2 years after TB infection is the same whether it is after a first infection or after re-infection

** Transfer out considered equivalent to default [91]. Overall cure rate if default based on timing of default [73], and cure rates from trials of very short course treatment [81-83].

Model cohort members were classified into five broad TB-related health states: 1) uninfected; 2) latent tuberculosis infection (LTBI); 3) active tuberculosis; 4) successfully treated, or spontaneously resolved active TB; and 5) chronic TB (MDR-TB). Latent TB, active TB, and spontaneously resolved active TB were further subdivided into three categories based on drug sensitivity: drug sensitive, single-drug resistant, or multi-drug resistant. The annual risk of infection was calculated from the estimated incidence of smear-positive cases using the Styblo formula [16]. We estimated that a new TB infection would progress to active disease in 5% of HIV-negative individuals within 2 years of acquiring the infection [17], and long-standing LTBI would re-activate at an annual rate of 0.1% thereafter in HIV-negative individuals [18,19]. To date, control strategies in low- and middle- income countries have focused largely on active cases, with limited treatment of latent infection [5]. We therefore assumed that the only persons treated for latent TB infection would be individuals diagnosed with concurrent HIV-latent TB infection (the latter based on tuberculin test results) [20]. Of those treated, we estimated only 67% would complete treatment for latent infection [21].

HIV-related health states were divided into uninfected, early HIV (asymptomatic), and late HIV (clinical AIDS). The annual risk of HIV infection was estimated to be 0.96%, calculated from the adult population prevalence of HIV in Zambia divided by the mean survival with HIV infection in a low-income setting [10,22]. The risks of TB progression, re-activation, relapse, and mortality were assumed to be much higher in HIV-positive individuals [23-26]. These and other key pathogenetic assumptions with respect to TB and HIV acquisition, progression, and clinical outcomes are listed in Table 2.

**Figure 1:**
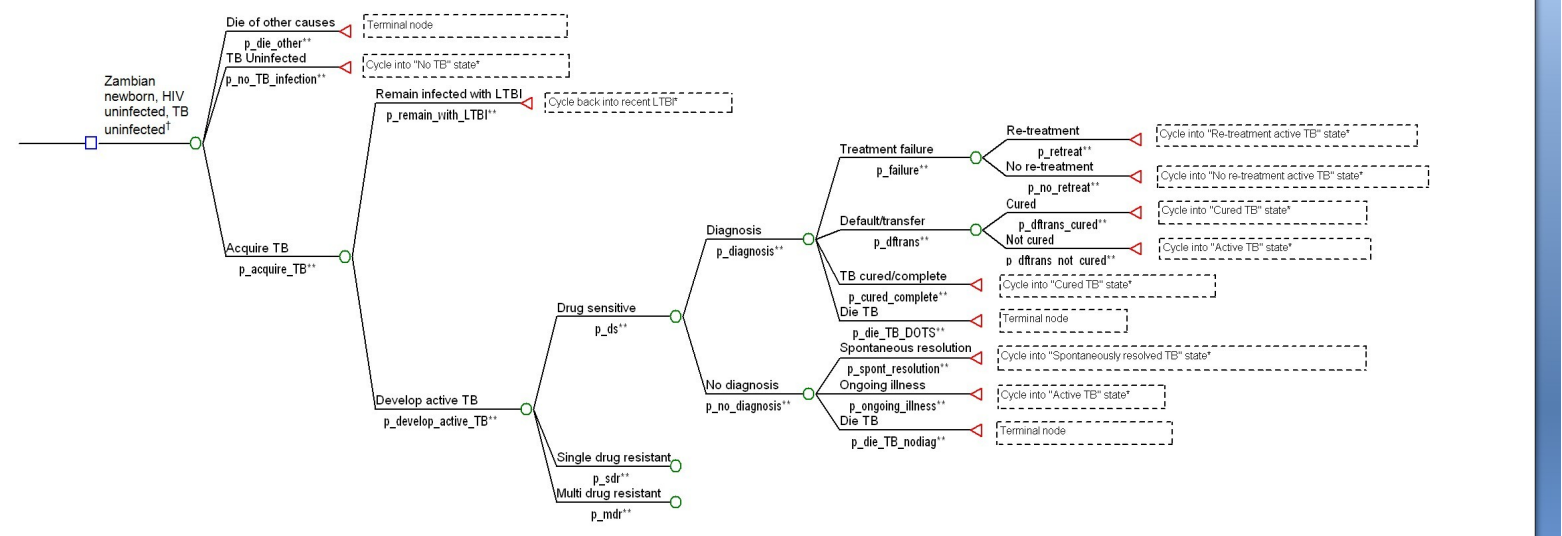
**Sample decision analysis tree for Zambian newborn initially without HIV or TB infection**. Footnotes: * States entered in subsequent cycles not shown in this figure ** The letter "p" preceding a variable name denotes probability † Decision tree structure for HIV infection not shown in this figure

Beginning from the first year, we estimated the proportion of the cohort developing active TB, dying from TB, dying from HIV, and dying from all other causes. In each year, costs associated with each branch of the decision tree were determined and accumulated. Cohort members surviving each year entered the subsequent year of the simulation, starting in the health state determined by events of the preceding year. Final outcomes including costs were multiplied by the size of the newborn population to generate expected values over the 30-year simulation, for the cohort of newborns born in Year 1. A simplified schematic of the decision tree is shown in Figure 1.

### Costs

Costs, expressed in 2007 U.S. dollars, were estimated from a societal perspective. Direct costs included those borne by the government and the healthcare system, plus costs for implementation and maintenance. Indirect costs included out-of-pocket expenditures by patients and families, and lost productivity due to TB related disability and death. For children with TB, indirect costs reflected costs borne by their families, namely family members taking time off work and/or paying out-of-pocket costs related to children's illness and care. A summary of direct and indirect costs per patient managed in Zambia is shown in Table 3.

**Table 3:** Direct and indirect costs per tuberculosis patient managed in Zambia

Type of Cost	Mean	Source
**Pre-Diagnosis**		
**Number of medical visits**	1.94	ZCQ^†^
Lab costs (3 AFB smears)	$8.49	[92]
Patient out-of-pocket expenditures for visits	$10.63	ZCQ^†^
**Indirect **Lost income for patient/family for visits*	$1.47	ZCQ^†^

**Post-Diagnosis**		
**Hospitalisation **Number of hospital days	7.13	ZCQ^†^
**Direct **Health system costs for hospital days	$98.68	HCQ^‡^
Patient out-of-pocket expenditures: hospital days	$128.91	ZCQ^†^
**Indirect **Lost income for patient/family for hospital days*	$15.61	ZCQ^†^

**Direct Observation of Treatment (DOT)**		
**Number of visits**	108	ZCQ^†^
**Direct **Health system costs for visits	$78.84	HCQ^‡^
Drug costs (new case)	$5.37	[33]
Patient out-of-pocket expenditures for visits	$15.12	ZCQ^†^
**Indirect **Lost income for patient/family for visits*	$51.84	ZCQ^†^

**Follow-up**		
**Number of visits**	7	ZCQ^†^
**Direct **Health system costs for visits	$27.44	HCQ^‡^
Patient out-of-pocket expenditures for visits	$0.84	ZCQ^†^
**Indirect **Lost income for patient/family for visits*	$4.06	ZCQ^†^

**Disability Costs**		
**Direct **Patient miscellaneous direct costs**	$2.12	ZCQ^†^
**Indirect **Lost income due to patient disability^¶^	$138.10	ZCQ^†^, HCQ^‡^
Family miscellaneous indirect costs^¶¶^	$46.44	HCQ^†^

**Total Cost per TB patient managed**		
**Direct **Health system	$226.43	
Patient out-of-pocket and miscellaneous costs	$157.62	
**Indirect **Patient/family lost income and miscellaneous costs	$257.52	
**Total **Health system and patient/family	$641.57	

**Vaccination**		
Initial investment^§ ^- BCG replacement only	$1.20/vaccinated	[7,34,57]
With booster (total)	$1.65/vaccinated	[7,34,57]
Unit cost^§§ ^- BCG	$2.00	[93]
BCG replacement only	$1.10 ^††^	[9]
With Booster (total)	$3.40	[9]

† Values based on Zambian TB Cost Questionnaire 2006 [27]

‡ Values based on Haiti TB Patient Cost Questionnaire [15], where GNI per capita is similar to Zambia

* Lost income based on average per capita gross national income - $500 - [58] and average work week of 40 hours

** Direct patient/family expenditures (while ill with TB disease) that are in addition to money spent while visiting health establishments or hospitals (e.g. cleaning, food supplements, childcare)

¶ Disability costs based on cure/complete treatment outcome (2 months disability) [28-30]

¶¶ Based on total hours spent by family members helping patient at home

§ Initial investment includes research, development, and production costs

§§ Unit cost includes distribution, administration, and vaccine dose costs.

†† BCG replacement unit cost estimates provided by Aeras [9] were lower than that of current BCG - likely secondary to improved manufacturing and/or distribution efficiency

Data regarding the number of health care visits and out-of-pocket expenditures for patients and families in the pre-diagnostic, hospitalization, treatment, and follow-up phases were collected and analyzed from a previous survey of adult TB patients in Zambian urban primary health care centres [27]. As we did not have directly gathered data for costs to the Zambian health care system, health care system costs were estimated from a survey of Haitian TB care providers [15], where the annual gross national income (GNI) per capita at the time of the study in 2003 was similar to that of Zambia now. Lost income due to TB-related medical visits and TB-related premature death were determined by the model based on GNI per capita, the number of remaining years in the simulation, and the presence or absence of HIV infection. The median length of survival for HIV-infected persons was estimated to be 9.8 years with early HIV and 9 months with clinical AIDS [22]. For adults with active TB. disability costs were calculated based on an assumed 50% reduction in productivity from symptom onset until diagnosis, and during the first 2 months of treatment [28-30]. Untreated patients or patients who failed treatment had a 50% reduction in productivity for the duration of their illness.

Costs for the maintenance of the DOTS program were based on detailed evaluations of DOTS implementation in Ecuador [31], adjusted for gross national income per capita in Zambia and converted to 2007 dollars using the consumer price index [32]. Costs of anti-tuberculosis drugs for active and latent TB were as listed by the Global Drug Facility [33].

Initial research, development, and production costs for single vaccination and prime-boost strategies were projected to be $141 million and $194 million respectively, and prorated to the Zambian share (0.4%) of global BCG vaccine coverage for newborns [34]. Survey data collected prior to the 36^th ^IUATLD Conference in Paris (2005) provided the basis for all vaccine costs [9], for which the Aeras 403 recombinant BCG (rBCG) was used as the replacement TB vaccine, and the Aeras 402 Crucell Ad35 vector expressing TB antigens was used as the booster. Of note, we assumed no additional infrastructure costs for distribution and delivery of a novel vaccine, as an extensive program for BCG vaccination already exists as part of the WHO Expanded Program for Immunization (EPI). We assumed that any HIV screening costs associated with vaccination would be the same as for the current BCG program.

### Sensitivity analyses

We performed extensive sensitivity analyses to test the robustness of the model to a range of parameter values. Key epidemiologic factors such as HIV prevalence and TB infection risk were varied, where available, within published ranges. Assumed costs were doubled and quadrupled to assess their impact on the cost effectiveness of the vaccine strategies. To account for potentially higher discount rates in low-income countries, we considered discount rates ranging from 2-6%. In addition, combinations of unfavorable assumptions were used to describe a "worst case" scenario. Since the first new vaccines are now in the early and middle phases of human clinical trials, there are no definitive efficacy data. Therefore, we examined the effects of varying the assumed efficacy and mechanism of action for the vaccine strategies. We considered prevention of acquisition of initial infection, and prevention of late reactivation as alternate mechanisms for vaccine action.

This study used a hypothetical simulation model based on previously published data, so research ethics committee approval was not required. McGill University, the Fonds de la Recherche en Santé du Québec, and the Canadian Institutes of Health Research provided salary support to the researchers, but had no role in any aspect of this study. All members of the research team had full access to all data and to the decision analysis model.

## Results

With the status quo, the model projected 25,557 active TB cases and 18,379 TB-related deaths over a period of 30 years, among 468,073 Zambians born in Year 1. The associated direct costs were $11.4 million and indirect costs were $45.1 million. Relative to current TB control measures, a BCG replacement vaccine administered at birth, with 70% efficacy in preventing rapid progression to TB disease after initial infection, is estimated to prevent 932 active TB cases and 422 TB-related deaths over the same period. This corresponds to a reduction of 199 cases and 90 deaths per 100,000 vaccinated. The prevention of these active TB cases and TB-related deaths would decrease direct costs by $0.2 million, and indirect costs by $3.4 million, resulting in a net reduction of $3.6 million in societal costs. Hence a new vaccine is predicted to result in cost savings as well as reduced morbidity and mortality.

The addition of a booster vaccine dose would avert 1,863 active TB cases and 1,011 TB-related deaths as compared to the status quo--a further reduction of 931 TB cases and 589 TB-related deaths beyond a single neonatal dose of the new vaccine. However, this would increase total direct costs to $11.9 million, reflecting larger investment in research, development, distribution, and vaccine administration. Substantial indirect cost savings of $6.2 million however, would produce net societal cost savings of $5.6 million compared to the status quo, or $2.0 million compared to a single dose of the new vaccine at birth.

**Figure 2:**
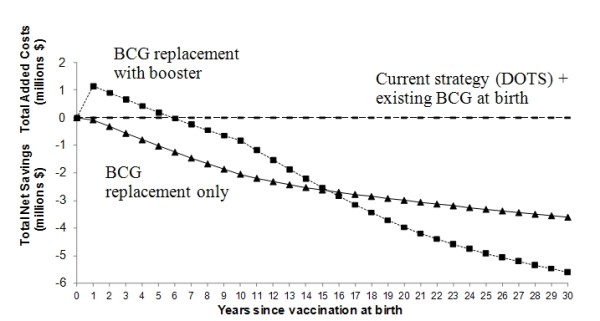
**Net savings or added cost of vaccine strategies over 30 years relative to status quo**.

Tables 4 and 5 summarize TB related costs, TB morbidity and mortality for each strategy over varying time horizons, for the entire group of newborns (Table 4), and per 100,000 newborns (Table 5). With vaccination at birth alone, net savings for Zambia would begin within 1 year, whereas the prime-boost strategy would require an additional 5 years to realize savings - reflecting greater initial development costs (Figure 2). In the long run (>16 years), the prime-boost vaccination strategy would be the cheapest.

**Table 4:** Cost and effectiveness of three strategies for tuberculosis control in Zambian newborns over 30 years, for the total cohort of 468,073 newborns

Strategies		Total Costs	Direct Costs	Indirect Costs	TB Cases	TB Mortality
Ranked from least to most expensive with respect to total cost		$ million	$ million	$ million		
**After 5 years**	Novel TB vaccine at birth	7.123	3.348	3.775	5,771	2,861
	Status quo	8.154	3.539	4.615	6,643	3,215
	Novel TB vaccine at birth with Booster	8.347	4.572	3.775	5,771	2,861

**After 10 years**	Novel TB vaccine at birth	16.702	5.721	10.981	11,608	6,452
	Novel TB vaccine at birth with Booster	17.926	6.945	10.981	11,608	6,452
	Status quo	18.754	5.980	12.774	12,592	6,896

**After 20 years**	Novel TB vaccine at birth with Booster	36.486	10.129	26.357	19,388	12,772
	Novel TB vaccine at birth	37.459	9.330	28.129	20,366	13,380
	Status quo	40.463	9.583	30.880	21,305	13,808

**After 30 years**	Novel TB vaccine at birth with Booster	50.876	11.930	38.946	23,694	17,368
	Novel TB vaccine at birth	52.863	11.126	41.737	24,625	17,957
	Status quo	56.481	11.377	45.104	25,557	18,379

**Table 5:** Cost and effectiveness of three strategies for tuberculosis control in Zambian newborns over 30 years, per 100,000 newborns.

Strategies		Total Costs	Direct Costs	Indirect Costs	TB Cases	TB Mortality
Ranked from least to most expensive with respect to total cost		$million	$million	$million		
**After 5 years**	Novel TB vaccine at birth	1.522	0.715	0.806	1,233	611
	Status quo	1.742	0.756	0.986	1,419	687
	Novel TB vaccine at birth with Booster	1.783	0.977	0.806	1,233	611

**After 10 years**	Novel TB vaccine at birth	3.568	1.222	2.346	2,480	1,378
	Novel TB vaccine at birth with Booster	3.830	1.484	2.346	2,480	1,378
	Status quo	4.007	1.278	2.729	2,690	1,473

**After 20 years**	Novel TB vaccine at birth with Booster	7.795	2.164	5.631	4,142	2,729
	Novel TB vaccine at birth	8.003	1.993	6.010	4,351	2,859
	Status quo	8.645	2.047	6.597	4,552	2,950

**After 30 years**	Novel TB vaccine at birth with Booster	10.869	2.549	8.320	5,062	3,711
	Novel TB vaccine at birth	11.294	2.377	8.917	5,261	3,836
	Status quo	12.067	2.431	9.636	5,460	3,927

### Sensitivity analyses

Table 6 summarizes sensitivity analyses for key parameters. Net cost savings related to prevention of additional TB cases would be expected even if the initial cost of research and development were quadrupled, or if the vaccine unit cost were quadrupled. If the vaccine's duration of action were halved to 5 years, BCG replacement vaccination at birth alone would produce a total added societal cost of $0.55 million, and prevent fewer TB cases than the current BCG vaccine, which had an assumed duration of action of 10 years. However, with the prime-boost vaccination strategy there were predicted societal cost savings even when the vaccine's duration of action was halved.

**Table 6:** Sensitivity analysis of vaccine strategies for tuberculosis control in Zambia, for the total cohort of 468,073 newborns

Parameter Varied	No. of TB cases prevented*	Change in Direct Costs† $ million	Change in Indirect Costs† $ million	Change in Total Costs† $ million
	
	All values are relative to the status quo strategy over 30 years			
**Novel TB Vaccine at Birth**				
Base case (no change in parameters)	932	($0.25)	($3.37)	($3.62)
Initial investment doubled	932	$0.31	($3.37)	($3.06)
Initial investment quadrupled	932	$1.44	($3.37)	($1.93)
Vaccine unit costs doubled	932	$0.24	($3.37)	($3.13)
Vaccine unit costs quadrupled	932	$1.20	($3.37)	($2.17)
Initial investment & vaccine unit costs doubled	932	$0.79	($3.37)	($2.58)
Initial investment & vaccine unit costs quadrupled	932	$2.89	($3.37)	($0.48)
Vaccine duration of action halved (5 yrs)	(98)	$0.22	$0.33	$0.55
Initial investment & vaccine unit costs doubled & vaccine duration of action halved	(98)	$1.26	$0.33	$1.59
HIV prevalence increased by 50%	1,020	($0.28)	($3.57)	($3.85)
HIV prevalence decreased by 50%	833	($0.23)	($3.16)	($3.39)
Annual risk of TB infection increased by 50%	1,297	($0.41)	($4.67)	($5.08)
Annual risk of TB infection decreased by 50%	501	($0.06)	($1.83)	($1.89)
Discount rate 2% annually	936	($0.26)	($3.79)	($4.05)
Discount rate 6% annually	899	($0.23)	($2.46)	($2.69)

**Novel TB Vaccine at Birth with Booster**				
Base case (no change in parameters)	1,863	$0.55	($6.16)	($5.61)
Initial investment doubled	1,863	$1.33	($6.16)	($4.83)
Initial investment quadrupled	1,863	$2.87	($6.16)	($3.29)
Vaccine unit costs doubled	1,863	$2.05	($6.16)	($4.11)
Vaccine unit costs quadrupled	1,863	$5.05	($6.16)	($1.11)
Initial investment & vaccine unit costs doubled	1863	$2.82	($6.16)	($3.34)
Initial investment & vaccine unit costs quadrupled	1,863	$7.36	($6.16)	$1.20
Vaccine duration of action halved (5 yrs)	904	$0.98	($3.18)	($2.20)
Initial investment & vaccine unit costs doubled & vaccine duration of action halved	904	$3.25	($3.18)	$0.07
HIV prevalence increased by 50%	1,994	$0.53	($6.48)	($5.95)
HIV prevalence decreased by 50%	1,718	$0.58	($5.81)	($5.23)
Annual risk of TB infection increased by 50%	2,294	$0.37	($7.64)	($7.27)
Annual risk of TB infection decreased by 50%	1,151	$0.87	($3.78)	($2.91)
Discount rate 2% annually	1,985	$0.59	($7.15)	($6.56)
Discount rate 6% annually	1,568	$0.45	($4.09)	($3.64)

*For TB cases prevented, values in parentheses indicate a net increase in TB cases

†For costs: values in parentheses indicate net savings, values without parentheses indicate net cost increases

Savings as well as prevention of morbidity and mortality would increase for both vaccination strategies if the prevalence of HIV were higher, reflecting the effect of concurrent HIV infection on risks of rapid primary TB progression and late TB re-activation. Similarly, if the annual risk of TB infection were higher, cost savings would increase. Conversely, with decreases in HIV prevalence or in the annual risk of TB infection, the number of TB cases prevented by vaccination would decrease. Nonetheless, net cost savings are expected with both vaccine strategies even with a 50% reduction in HIV prevalence or a 50% reduction in the annual risk of TB infection. With increases in the annual discount rate, projected cost savings decrease as do TB cases prevented and deaths prevented. Net cost savings are predicted for both vaccine strategies even when the discount rate is doubled, to 6%.

In a "worst case" scenario where three key assumptions were made less favorable (initial development cost doubled, vaccine unit cost doubled, vaccine duration of action halved), both vaccination strategies would be associated with increased societal costs as compared to the status quo strategy. For the single BCG replacement dose, 98 more TB cases and 87 more TB deaths would be expected, as well as higher societal costs. For the prime-boost strategy, additional societal costs would then be $77 per case prevented, and $160 per death averted, as compared to the status quo.

**Figure 3:**
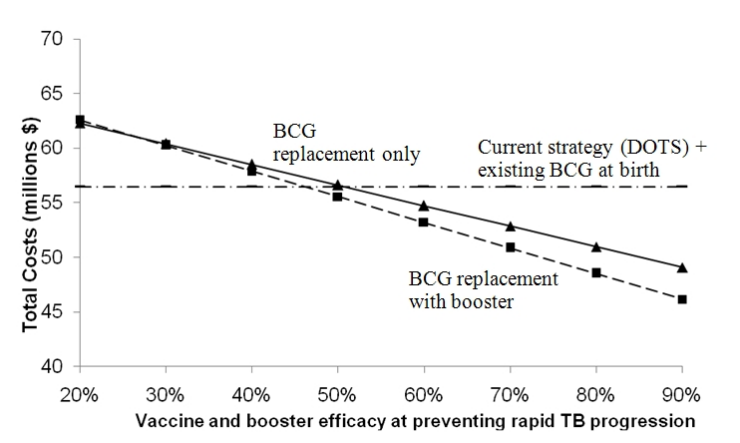
**Effect of varying vaccine and booster efficacy when mechanism is prevention of rapid TB progression**. Protective effect acting on rapid TB progression only - none at initial infection or re-activation - on the projected total costs over 30-years.

**Figure 4:**
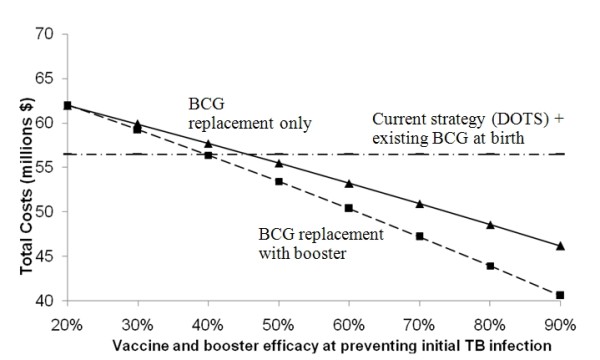
**Effect of varying vaccine and booster efficacy when mechanism is prevention of initial TB infection **Protective effect acting on initial TB infection only - none at rapid progression or re-activation - on the projected total costs over 30 years.

**Figure 5:**
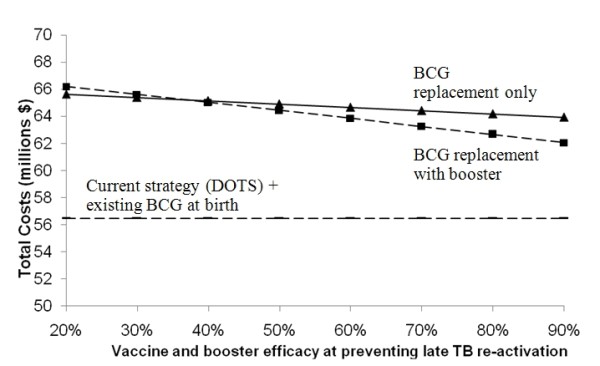
**Effect of varying vaccine and booster efficacy when mechanism is prevention of late TB re-activation**. Protective effect acting on late TB re-activation only - none at initial infection or rapid progression - on the projected total costs over 30 years.

Changing the predicted efficacy and assumed mechanism of action showed that vaccines with efficacy ≥60% and 50% targeting rapid progression and acquisition of infection respectively, would be cost-saving relative to current conditions (Figures 3 and 4). Due to the low annual risk of re-activation in HIV-negative persons (0.1%), the spreading of risk over a lifetime, and loss of partial protection against rapid primary progression conferred by the current BCG vaccine, a vaccine targeting late reactivation alone would be more costly than the status quo, even with 90% efficacy (Figure 5). Of course, a vaccine that is safe and effective in preventing late reactivation is likely to be widely administered throughout the community (not only to newborns), a scenario that was beyond the scope of the present analysis.

## Discussion

Our comparison of two vaccination strategies with the current DOTS and BCG vaccination strategy for tuberculosis control in Zambia suggest that improved vaccines could reduce TB-related mortality and morbidity, and produce cost savings over a 30-year time horizon. The projections were generally robust in sensitivity analyses, even with assumed protective efficacy of 60% with a novel vaccine. Not surprisingly, neonatal vaccines which target initial infection or rapid progression will be more cost-effective in the short-term than those which target late re-activation. The current pipeline for novel vaccines predominantly focuses on these mechanisms of action [8,35].

A previous analysis examined the public health impact of new tuberculosis vaccines and predicted that a pre-exposure vaccine, targeting the acquisition of infection or primary progression would be effective at preventing a substantial proportion of active TB cases. Although such a vaccine would not benefit persons who have already become infected with *Mycobacterium tuberculosis*, it would nonetheless provide important public health gains with respect to tuberculosis-related morbidity and mortality. The authors predicted that a pre-exposure vaccine that acts solely by reducing later reactivation of latent infection would not have the same impact, even if the vaccine were highly efficacious [36]. Our findings are similar and concordant with another recent analysis that used different modeling methods [37]. Over the long term, any vaccine that reduces late reactivation will decrease the annual risk of TB infection and therefore decrease disease incidence. However, the low annual risk of re-activation in HIV-negative persons, combined with finite vaccine duration of action make such a vaccine less cost-effective in the short or medium term.

The cost-effectiveness of a vaccine targeting late reactivation will be enhanced if it provides longer lasting immunity, and potentially if it is administered only after the acquisition of latent infection. One report estimated that a pre-exposure vaccine with efficacy of 50% - 90%, with a single mechanism of action, may only be capable of reducing TB morbidity by one third over the long term [36]. However, a more recent analysis concluded that a neonatal vaccine could reduce TB incidence by 39-55% in the long term, while a mass pre-exposure vaccine campaign could reduce incidence by 67%, in the absence of HIV infection [38]. The same analysis suggested that the combination of pre- and post- exposure vaccines could reduce TB incidence by 79% in Southeast Asia by 2050, in the absence of HIV infection. Hence, a TB control strategy that combines the DOTS program with vaccines targeting both early infection/progression and late reactivation may ultimately be considered.

The duration of vaccine-induced immunity remains unclear for the current BCG vaccine [6,39,40], so no concrete estimates exist for novel BCG replacement vaccines. For a vaccine with waning efficacy, a prime-boost strategy would be more cost-effective in the long term (Figure 2); however, the time lag to net savings may present a barrier to investment by both the public and private sectors.

A limitation of our analysis was the prorated attribution of the initial vaccine development, research, and production costs to Zambia, in proportion to its current share of global BCG vaccination coverage (0.398%). At this point in vaccine development there remains great uncertainty about the ultimate cost of research, pre-clinical and clinical trials, and vaccine rollout. It is quite conceivable that these costs will exceed the estimates used in our primary analysis. However, sensitivity analyses suggested cost savings for the neonatal replacement vaccine, even if the initial investment and vaccine unit costs were both quadrupled. Our prorating of development costs to a single cohort of newborns is also conservative, as it tends to underestimate the cost-effectiveness of TB vaccines over time.

In fact, high-incidence low- and middle- income countries which will use novel TB vaccines may bear little of their initial development costs. The funds may be provided primarily by governments of high-income countries, non-governmental organizations and research grants. This means that from the perspective of high-incidence countries and their populations, novel tuberculosis vaccines will be even more cost-effective. On the other hand, from the broader global TB control perspective, which includes costs to funders, these costs are relevant to the present analysis.

We did not model potential adverse events related to the administration of tuberculosis vaccines in neonates. An open-label, phase I trial of a novel vaccine candidate has demonstrated safety and high immunogenicity in individuals with latent tuberculosis infection [41]. The frequency of disseminated BCG has been reported to be less than five per million, and is mainly associated with congenital immunocompromised states [42]. A recent revision to the WHO guidelines on BCG vaccination recommends the immunization of asymptomatic infants whose HIV status is unknown, but advises against immunization of 1) infants whose HIV status is unknown, but display signs or symptoms suggestive of HIV infection; and 2) HIV positive infants, regardless of signs or symptoms [43]. A previous study showed that the risk of disseminated BCG disease is increased several hundredfold in HIV-infected infants compared to the documented risk in HIV-uninfected infants [44]. Unfortunately, due to the transplacental passage of maternal HIV antibodies, accurate diagnosis in the neonatal period requires the demonstration of HIV DNA, or HIV RNA and p24 antigen. Furthermore, signs of HIV infection are uncommon prior to BCG vaccine administration in the first weeks of life. For these reasons, the current program for BCG vaccination may have reduced efficacy in a particularly high-risk group, and increased morbidity, mortality, and added costs may result from BCG-related adverse events in countries with high HIV prevalence such as Zambia. Conversely, safety in HIV-infected neonates is considered a prerequisite for any future neonatal TB vaccine.

Other potential limitations of the analysis included our assumptions of stability in population size and age distribution, and HIV parameters. Herd immunity was not modeled [45], but a reduction in the annual risk of TB infection resulting from any vaccine which prevents contagious pulmonary TB would further reduce TB morbidity and mortality. Similarly, we did not model transmission, since the primary analysis considered vaccine protection among children, in whom TB is rarely contagious. Again, this makes our model conservative in that it tends to underestimate potential vaccine benefits.

A challenge to TB vaccine development is the lack of proven immunological correlates of vaccine-induced immunity, although early animal studies suggest improved protection against a pulmonary TB challenge for several prime-boost approaches [46-48]. We did not model the effects of *M. tuberculosis *strain diversity. A recent analysis suggests the efficacy of novel vaccines may differ against different strains of the bacteria [49], however, the precise impact on efficacy remains uncertain given our incomplete knowledge regarding how distinct strains interact and the strain specificity of current vaccine candidates.

Our findings remained consistent across different scenarios for HIV seroprevalence. Greater savings and reduction in TB-related morbidity and mortality would be achieved if HIV seroprevalence was higher, owing to the more frequent development of active TB following infection. An increase in the proportion of TB which is multi-drug resistant would yield greater cost savings for vaccination, because of increased treatment and patient/family costs for MDR-TB. We computed the annual risk of TB infection from the incidence of smear-positive cases using the Styblo formula, which has been criticized [50]. Furthermore, a recent study has shown that in the presence of a strong control program, the TB incidence is not necessarily reflective of the true annual risk of TB infection in a country with high HIV seroprevalence [51]. However, we substantially varied the assumed annual risk of TB infection, and demonstrated that both primary vaccination strategies considered would still result in cost savings. Any inaccuracy in estimating the annual risk of TB infection would apply equally to all strategies, and would not tend to favor one particular strategy over the others.

Health care system costs were approximated by previously published data in Haiti, where GNI per capita is similar to Zambia. Social, political, and economic differences between the two countries may affect the validity of these estimates. However, indirect costs, which were obtained from Zambia itself, accounted for the bulk of total societal costs, strengthening our cost estimates. As a case study, our results may not be directly applicable to other sub-Saharan African countries given variability in epidemiology and economics, but the flexibility of the decision analysis model suggests the potential for a similar approach.

Several strengths of the analysis deserve comment. We considered the effect of HIV infection on TB pathogenesis, and accounted for survival with asymptomatic HIV and clinical AIDS, since HIV prevalence is high in Zambia and other sub-Saharan African countries [10]. Published values were used for epidemiologic data, TB pathogenesis, HIV pathogenesis, and treatment outcomes whenever available, thereby reducing uncertainty. We evaluated costs from a societal perspective, and considered both direct and indirect costs from the standpoint of a low-income country with high TB incidence. Notably, indirect costs borne by patients and families constituted a substantial proportion of the total. The decision analysis model allowed us to manipulate key variables and test our underlying assumptions. Sensitivity analyses confirmed the robustness of the main findings. In particular, with an assumed efficacy of 60%, a novel vaccine that prevents rapid progression to active TB would still provide cost savings, as well as prevent TB cases and TB-related deaths. This strengthens the economic argument in favour of developing and implementing new vaccines. We recognize that the 70% efficacy estimate used for our primary analysis is speculative, as useful evidence must await the outcome of one or more large-scale clinical trials, still years away.

Novel TB vaccine development and deployment faces a number of obstacles. The lack of validated biomarkers for candidate vaccine selection, scarcity of suitable field sites, exclusion of at-risk populations from trials, regulatory issues, and complex ethical concerns at each stage of human testing are among the challenges [52-54]. The continued expansion of the TB vaccine pipeline, however, remains reassuring. Recent years have renewed interest in TB vaccine development. By the end of 2009, at least nine novel TB vaccines were undergoing evaluation in humans, with at least two recombinant protein vaccines reaching phase II trials [54,55].

## Conclusions

This analysis suggests that a prime-boost strategy using a tuberculosis vaccine with moderate efficacy (>60%) which prevents initial infection or rapid primary progression to disease, will be the most cost-effective vaccine intervention over the short to medium term, in high-burden countries. We conclude that investment in an improved TB vaccine may result in considerable cost savings, as well as a reduction in TB morbidity and TB-related mortality, when it enhances existing control strategies.

## Competing interests

The authors declare that they have no competing interests.

## Authors' contributions

C-LT aided in design of the study, acquired study data, conducted the primary analyses, and wrote the first draft of the manuscript. OO aided in design of the study and acquisition of data, aided the analyses, and provided critical revisions to the manuscript. DM aided in design of the study and in acquisition and analysis of data, and provided critical revisions to the manuscript. AA aided in study design, acquisition and analysis of data, and provided critical revisions to the manuscript. KS supervised study design, data acquisition and analysis, and helped draft and critically revise the manuscript. All authors have read and approved the final manuscript.

## Pre-publication history

The pre-publication history for this paper can be accessed here:

http://www.biomedcentral.com/1471-2458/11/55/prepub

## References

[B1] Global tuberculosis control: epidemiology, strategy, financing WHO report 2009 2009 Geneva: WHO Press

[B2] Meulemans H Mortelmans D Liefooghe R Mertens P Zaidi SA Solangi MF De Muynck A The limits to patient compliance with directly observed therapy for tuberculosis: a socio-medical study in Pakistan Int J Health Plann Manage 2002 17 249 267 10.1002/hpm.675 12298146

[B3] Elzinga G Raviglione MC Maher D Scale up: meeting targets in global tuberculosis control Lancet 2004 363 814 819 10.1016/S0140-6736(04)15698-5 15016493

[B4] Bonnet M Sizaire V Kebede Y Janin A Doshetov D Mirzoian B Arzumanian A Muminov T Iona E Rigouts L Rüsch-Gerdes S Varaine F Does one size fit all? Drug resistance and standard treatments: results of six tuberculosis programmes in former Soviet countries Int J Tuberc Lung Dis 2005 9 1147 1154 16229227

[B5] Global Plan to Stop TB 2006-2015 2006 Stop TB Partnership and World Health Organization. Geneva, World Health Organization (WHO/HTM/STB/2006.35) 10.2471/06.038513 PMC2636638 17639210

[B6] Colditz GA Berkey CS Mosteller F Brewer TF Wilson ME Burdick E Fineberg HV The efficacy of bacillus Calmette-Guérin vaccination of newborns and infants in the prevention of tuberculosis: meta-analysis of the published literature Pediatrics 1995 96 29 35 7596718

[B7] Rodrigues LC Diwan VK Wheeler JG Protective effect of BCG against tuberculous meningitis and military tuberculosis: a meta-analysis Int J Epidemiol 1993 22 1154 1158 10.1093/ije/22.6.1154 8144299

[B8] Skeiky YA Sadoff JC Advances in tuberculosis vaccine strategies Nat Rev Microbiol 2006 4 469 476 10.1038/nrmicro1419 16710326

[B9] Menzies D A survey of vaccine technology Presentation at 36th Union World Conference on lung health, Paris, France 2005

[B10] UNAIDS 2006 Report on the global AIDS epidemic http://www.unaids.org/en/KnowledgeCentre/HIVData/GlobalReport/2006/default.asp

[B11] Weinstein MC Siegel JE Gold MR Kamlet MS Russell LB Recommendations of the Panel on cost-effectiveness in health and medicine JAMA 1996 276 1253 1258 10.1001/jama.276.15.1253 8849754

[B12] Brewer TF Preventing tuberculosis with bacillus Calmette-Guérin vaccine: a meta-analysis of the literature Clin Infect Dis 2000 31 Suppl 3 64 67 10.1086/314072 11010824

[B13] Colditz GA Brewer TF Berkey CS Wilson ME Burdick E Fineberg HV Mosteller F Efficacy of BCG vaccine in the prevention of tuberculosis. Meta-analysis of the published literature JAMA 1994 271 698 702 10.1001/jama.271.9.698 8309034

[B14] Schwartzman K Oxlade O Barr RG Grimard F Acosta I Baez J Ferreira E Melgen RE Morose W Salgado AC Jacquet V Maloney S Laserson K Mendez AP Menzies D Domestic returns from investment in the control of tuberculosis in other countries N Engl J Med 2005 353 1008 1020 10.1056/NEJMsa043194 16148286

[B15] Jacquet V Morose W Schwartzman K Oxlade O Barr G Grimard F Menzies D Impact of DOTS expansion on tuberculosis related outcomes and costs in Haiti BMC Public Health 2006 6 209 10.1186/1471-2458-6-209 16911786 PMC1590025

[B16] Styblo K The relationship between the risk of tuberculosis infection and the risk of developing infectious tuberculosis Bull Int Union Tuberc 1985 60 117 119

[B17] Sutherland I The evolution of clinical tuberculosis in adolescents Tuberc 1966 47 308

[B18] Nolan CM Elarth AM Tuberculosis in a cohort of Southeast Asian refugees. A five-year surveillance study Am Rev Respir Dis 1988 137 805 809 3354985 10.1164/ajrccm/137.4.805

[B19] Comstock GW Edwards LB Livesay VT Tuberculosis morbidity in the U.S. Navy: its distribution and decline Am Rev Respir Dis 1974 110 572 580 4429253 10.1164/arrd.1974.110.5.572

[B20] Burgess AL Fitzgerald DW Severe P Joseph P Noel E Rastogi N Johnson WD Jr Pape JW Integration of tuberculosis screening at an HIV voluntary counselling and testing centre in Haiti AIDS 2001 15 1875 79 10.1097/00002030-200109280-00018 11579251

[B21] Dasgupta K Schwartzman K Marchand R Tannenbaum TN Brassard P Menzies D Comparison of cost effectiveness of tuberculosis screening of close contacts and foreign-born populations Am J Respir Crit Care Med 2000 162 2079 2086 11112118 10.1164/ajrccm.162.6.2001111

[B22] Morgan D Mahe C Mayanja B Okongo JM Lubega R Whitworth JA HIV-1 infection in rural Africa: is there a difference in median time to AIDS and survival compared with that in industrialized countries? AIDS 2002 16 597 603 10.1097/00002030-200203080-00011 11873003

[B23] Wood R Maartens G Lombard CJ Risk factors for developing tuberculosis in HIV-1-infected adults from communities with low or very high incidence of tuberculosis J Acquir Immune Defic Syndr 2000 23 75 80 10708059 10.1097/00126334-200001010-00010

[B24] Murray J Sonnenberg P Shearer SC Godgrey-Faussett P Human immunodeficiency virus and outcome of treatment for new and recurrent pulmonary tuberculosis in African patients Am J Respir Crit Care Med 1999 159 733 740 10051244 10.1164/ajrccm.159.3.9804147

[B25] Edlin BR Tokars JI Grieco MH Crawford JT Williams J Sordillo EM Ong KR Kilburn JO Dooley SW Castro KG Jarvis WR Holmberg SD An outbreak of multi-drug resistant tuberculosis among hospitalized patients with the acquired immunodeficiency syndrome New Engl J Med 1992 326 1514 1521 10.1056/NEJM199206043262302 1304721

[B26] Malkin JE Prazuck T Simonnet F Yameogo M Rochereau A Ayerour J Masson D Lafaix C Tuberculosis and human immunodeficiency virus infection in West Burkina Faso: clinical presentation and clinical evolution Int J Tuberc Lung Dis 1997 1 68 74 9441062

[B27] Aspler A Menzies D Oxlade O Banda J Mwenge L Godfrey-Faussett P Ayles H Cost of tuberculosis diagnosis and treatment from the patient perspective in Lusaka, Zambia Int J Tuberc Lung Dis 2008 12 928 935 18647453

[B28] Sherman LF Fujiwara PI Cook SV Bazerman LB Frieden TR Patient and health care system delays in the diagnosis and treatment of tuberculosis Int J Tuberc Lung Dis 1999 3 1088 1095 10599012

[B29] Yamasaki-Nakagawa M Ozasa K Yamada N Osuga K Shimouchi A Ishikawa N Bam DS Mori T Gender difference in delays to diagnosis and health care seeking behaviour in a rural area of Nepal Int J Tuberc Lung Dis 2001 5 24 31 11263512

[B30] Wandwalo ER Morkve O Delay in tuberculosis case-finding and treatment in Mwanza, Tanzania Int J Tuberc Lung Dis 2000 4 133 138 10694091

[B31] Vaca J Peralta H Gresely L Cordova R Kuffo D Romero E Tannenbaum TN Houston S Graham B Hernandez L Menzies D DOTS implementation in a middle-income country: development and evaluation of a novel approach Int J Tuberc Lung Dis 2005 9 521 527 15875923

[B32] U.S. Department of Labor Statistics Consumer price index 2007 http://www.stats.bls.gov

[B33] Stop TB Partnership Global Drug Facility Stop TB Partnership First-Line tuberculosis drugs & formulations currently supplied/to be supplied by the global TB drug facility, Geneva 2007 http://www.stoptb.org/gdf/

[B34] World Health Organization Vaccine Preventable Diseases Monitoring System 2007 http://www.who.int/vaccines/globalsummary/immunization/countryprofileselect.cfm

[B35] Baumann S Nasser Eddine A Kaufmann SH Progress in tuberculosis vaccine development Curr Opin Immunol 2006 18 438 448 Epub 2006 Jun 13 10.1016/j.coi.2006.05.016 16777396

[B36] Ziv E Daley CL Blower S Potential public health impact of new tuberculosis vaccines Emerg Infect Dis 2004 10 1529 1535 15498152 10.3201/eid1009.030921 PMC3320317

[B37] Bhunu CP Garira W Mukandavire Z Magombedze G Modelling the effects of pre-exposure and post-exposure vaccines in tuberculosis control Journal of Theoretical Biology 2008 254 633 649 10.1016/j.jtbi.2008.06.023 18644386

[B38] Abu-Raddad LJ Sabatelli L Achterberg JT Sugimoto JD Longini IM Jr Dye C Halloran ME Epidemiological benefits of more-effective tuberculosis vaccines, drugs, and diagnostics Proc Natl Acad Sci USA 2009 106 13980 13985 Epub 2009 Aug 3 10.1073/pnas.0901720106 19666590 PMC2720405

[B39] Aronson NE Santosham M Comstock GW Howard RS Moulton LH Rhoades ER Harrison LH Long-term efficacy of BCG vaccine in American Indians and Alaska Natives: A 60-year follow-up study JAMA 2004 291 2086 2091 10.1001/jama.291.17.2086 15126436

[B40] Fine PE Variation in protection by BCG: implications of and for heterologous immunity Lancet 1995 346 1339 1345 10.1016/S0140-6736(95)92348-9 7475776

[B41] Sander CR Pathan AA Beveridge NE Poulton I Minassian A Alder N Hill AV McShane H Safety and immunogenicity of a new tuberculosis vaccine, MVA85A, in *Mycobacterium *tuberculosis-infected individuals Am J Respir Crit Care Med 2009 179 724 733 10.1164/rccm.200809-1486OC 19151191 PMC2858810

[B42] Casanova JL Jouanguy E Lamhamedi S Blanche S Fischer A Immunological conditions of children with BCG disseminated infection Lancet 1995 346 581 10.1016/S0140-6736(95)91421-8 7658805

[B43] WHO Revised BCG vaccination guidelines for infants at risk for HIV infection Weekly Epidemiological Record 2007 82 193 196 17526121

[B44] Hesseling AC Marais BJ Gie RP Schaaf HS Fine PE Godfrey-Faussett P Beyers N The risk of disseminated Bacille Calmette-Guerin (BCG) disease in HIV-infected children Vaccine 2007 25 14 18 Epub 2006 Aug 1 10.1016/j.vaccine.2006.07.020 16959383

[B45] Brisson M Edmunds WJ Economic evaluation of vaccination programs: The impact of herd-immunity Med Decis Making 2003 23 76 82 10.1177/0272989X02239651 12583457

[B46] Brooks JV Frank AA Keen MA Bellisle JT Orme IM Boosting vaccine for tuberculosis Infect Immun 2001 69 2714 2717 10.1128/IAI.69.4.2714-2717.2001 11254639 PMC98211

[B47] Goonetilleke NP McShane H Hannan CM Anderson RJ Brookes RH Hill AV Enhanced immunogenicity and protective efficacy against *Mycobacterium *tuberculosis of bacille Calmette-Guérin vaccine using mucosal administration and boosting with a recombinant modified vaccinia virus Ankara J Immunol 2003 171 1602 1609 12874255 10.4049/jimmunol.171.3.1602

[B48] Wang J Thorson L Stokes RW Santosuosso M Huygen K Zganiacz A Hitt M Xing Z Single mucosal, but not parenteral, immunization with recombinant adenoviral-based vaccine provides potent protection from pulmonary tuberculosis J Immunol 2004 173 6357 6365 15528375 10.4049/jimmunol.173.10.6357

[B49] Cohen T Colijn C Murray M Modeling the effects of strain diversity and mechanisms of strain competition on the potential performance of new tuberculosis vaccines PNAS 2008 105 16302 16307 10.1073/pnas.0808746105 18849476 PMC2570977

[B50] van Leth F van der Werf MJ Borgdorff MW Prevalence of tuberculous infection and incidence of tuberculosis: a re-assessment of the Styblo rule Bull World Health Organ 2008 86 20 26 10.2471/BLT.06.037804 18235886 PMC2647347

[B51] Egwaga SM Cobelens FG Muwinge H Verhage C Kalisvaart N Borgdorff MW The impact of the HIV epidemic on tuberculosis transmission in Tanzania AIDS 2006 20 915 921 10.1097/01.aids.0000218557.44284.83 16549977

[B52] Fletcher HA Hawkridge T McShane H A new vaccine for Tuberculosis: The challenges of development and deployment J Bioeth Inq 2009 6 219 228 10.1007/s11673-009-9153-6 19536332 PMC2694314

[B53] Wallis RS Wang C Doherty TM Onyebujoh P Vahedi M Laang H Olesen O Parida S Zumla A Biomarkers for tuberculosis disease activity, cure, and relapse Lancet Infect Dis 2009 9 162 72 10.1016/S1473-3099(09)70042-8 19246020

[B54] Aagaard C Dietrich J Doherty M Andersen P TB vaccines: current status and future perspectives Immunol Cell Biol 2009 87 279 286 Epub 2009 Apr 7 19350048 10.1038/icb.2009.14

[B55] Working Group on new TB Vaccines Stop TB Partnership TB Vaccine Pipeline 2009 http://www.stoptb.org/wg/new_vaccines/assets/documents/TB%20Vaccine%20Pipeline%2009%20final.pdf

[B56] United Nations Populations Division World population prospects: the 2006 revision http://esa.un.org/unpp/

[B57] CIA - The World Factbook The World Factbook. Zambia 2007 https://www.cia.gov/library/publications/the-world-factbook/geos/za.html

[B58] The World Bank Group Zambia Data Profile 2007 http://web.worldbank.org/WBSITE/EXTERNAL/COUNTRIES/AFRICAEXT/ZAMBIAEXTN/0,,menuPK:375673~pagePK:141159~piPK:141110~theSitePK:375589,00.html

[B59] World Health Organization Life tables for WHO member states 2007 http://www.who.int/whosis/database/life_tables/life_tables.cfm

[B60] Anti-tuberculosis drug resistance in the world WHO report 2004, Geneva 2004

[B61] Zignol M Hosseini MS Wright A Weezenbeek CL Nunn P Watt CJ Williams BG Dye C Global incidence of multidrug-resistant tuberculosis J Infect Dis 2006 194 479 485 10.1086/505877 16845631

[B62] Deschamps MM Fitzgerald DW Pape JW Johnson WD Jr HIV infection in Haiti: natural history and disease progression AIDS 2000 14 2515 2521 10.1097/00002030-200011100-00014 11101063

[B63] Grzybowski S Barnett GD Styblo K Contacts of cases of active pulmonary tuberculosis Bull Int Union Tuberc 1975 50 90 106 1218291

[B64] Menzies D Issues in the management of contacts of patients with active pulmonary tuberculosis Can J Public Health 1997 88 197 201 9260361 10.1007/BF03403887 PMC6990350

[B65] Stead WW Management of health care workers after inadvertent exposure to tuberculosis: a guide for the use of preventive therapy Ann Intern Med 1995 122 906 12 7755225 10.7326/0003-4819-122-12-199506150-00003

[B66] Whalen CC Johnson JL Okwera A Hom DL Huebner R Mugyenyi P Mugerwa RD Ellner JJ A trial of three regimens to prevent tuberculosis in Ugandan adults infected with the human immunodeficiency virus. Uganda-Case Western Reserve University Research Collaboration Engl J Med 1997 337 801 808 10.1056/NEJM199709183371201 9295239

[B67] Guelar A Gatell JM Verdejo J Podzamczer D Lozano L Aznar E Miro JM Mallolas J Zamora L Gonzalez J Sorano E A prospective study of the risk of tuberculosis among HIV-infected patients AIDS 1993 7 1345 1349 10.1097/00002030-199310000-00007 8267907

[B68] Beck-Sagué C Dooley SW Hutton MD Otten J Breeden A Crawford JT Pitchenik AE Woodley C Cauthen G Jarvis WR Hospital outbreak of multidrug-resistant *Mycobacterium *tuberculosis infections. Factors in transmission to staff and HIV-infected patients JAMA 1992 268 1280 1286 1507374 10.1001/jama.1992.03490100078031

[B69] Fischl MA Uttamchandani RB Daikos GL Poblete RB Moreno JN Reyes RR Boota AM Thompson LM Cleary TJ Lai S An outbreak of tuberculosis caused by multiple-drug-resistant tubercle bacilli among patients with HIV infection Ann Intern Med 1992 117 177 183 1616211 10.7326/0003-4819-117-3-177

[B70] Small PM Shafer RW Hopewell PC Singh SP Murphy MJ Desmond E Sierra MF Schoolnik GK Exogenous reinfection with multidrug-resistant *Mycobacterium *tuberculosis in patients with advanced HIV infection N Engl J Med 1993 328 1137 1144 10.1056/NEJM199304223281601 8096066

[B71] Daley CL Small PM Schecter GF Schoolnik GK McAdam RA Jacobs WR Jr Hopewell PC An outbreak of tuberculosis with accelerated progression among persons infected with the human immunodeficiency virus: An analysis using restriction-fragment-length polymorphisms N Engl J Med 1992 326 231 235 10.1056/NEJM199201233260404 1345800

[B72] Grzybowski S Enarson DA The fate of cases of pulmonary tuberculosis under various treatment programmes Bull Int Union Tuberc 1978 53 70 74 737353

[B73] Horwitz O Public health aspects of relapsing tuberculosis Am Rev Respir Dis 1969 99 183 193 5767002 10.1164/arrd.1969.99.2.183

[B74] Reider HL Epidemiologic basis of tuberculosis control 1999 First Paris, France, International Union Against Tuberculosis and Lung Disease 1 162

[B75] Cohn DL Catlin BJ Peterson KL Judson FN Sbarbaro JA A 62-dose, 6-month therapy for pulmonary and extrapulmonary tuberculosis. A twice-weekly, directly observed, and cost-effective regimen Ann Intern Med 1990 112 407 415 2106816 10.7326/0003-4819-76-3-112-6-407

[B76] East African Tuberculosis Investigation Centre, British Medical Research Council Tuberculosis and Chest Diseases Unit Results at 5 years of a controlled comparison of a 6-month and a standard 18-month regimen of chemotherapy for pulmonary tuberculosis Am Rev Respir Dis 1977 116 3 8 69411 10.1164/arrd.1977.116.1.3

[B77] Somner AR Short-course chemotherapy in pulmonary tuberculosis. A controlled trial by the British Thoracic Association (third report) Lancet 1980 1 1182 1183 6103997

[B78] Algerian Working Group, British Medical Research Council Controlled clinical trial comparing a 6-month and a 12-month regimen in the treatment of pulmonary tuberculosis in the Algerian Sahara Am Rev Respir Dis 1984 129 921 928 6375490 10.1164/arrd.1984.129.6.921

[B79] Benator D Bhattacharya M Bozeman L Burman W Cantazaro A Chaisson R Gordin F Horsburgh CR Horton J Khan A Lahart C Metchock B Pachucki C Stanton L Vernon A Villarino ME Wang YC Weiner M Weis S Tuberculosis Trials Consortium Rifapentine and isoniazid once a week versus rifampicin and isoniazid twice a week for treatment of drug-susceptible pulmonary tuberculosis in HIV-negative patients: a randomised clinical trial Lancet 2002 360 528 534 10.1016/S0140-6736(02)09742-8 12241657

[B80] Chee CBE Boudville IC Chan SP Zee YK Wang YT Patient and disease characteristics, and outcome of treatment defaulters from the Singapore TB control unit - a one-year retrospective survey Int J Tuber Lung Dis 2000 4 496 503 10864179

[B81] Parthasarathy R Prabhakar R Somasundaram PR A controlled clinical trial of 3- and 5- month regimens in the treatment of sputum-positive pulmonary tuberculosis in South India Am Rev Respir Dis 1986 134 27 33 3524334 10.1164/arrd.1986.134.1.27

[B82] East African Tuberculosis Investigation Centre, British Medical Research Council Controlled clinical trial of five short-course (4-month) chemotherapy regimens in pulmonary tuberculosis: Second report of the 4th study Am Rev Respir Dis 1981 123 165 170 7015933 10.1164/arrd.1981.123.2.165

[B83] Singapore Tuberculosis Service, British Medical Research Council Long-term Follow-up of a clinical trial of six-month and four-month regimens of chemotherapy in the treatment of pulmonary tuberculosis Am Rev Respir Dis 1986 133 779 83 2871788

[B84] Espinal MA Kim SJ Suarez PG Kam KM Khomenko AG Migliori GB Baéz J Kochi A Dye C Raviglione MC Standard short-course chemotherapy for drug-resistant tuberculosis: treatment outcomes in 6 countries JAMA 2000 283 2537 2545 10.1001/jama.283.19.2537 10815117

[B85] Nathanson E Lambregts-van Weezenbeek C Rich ML Gupta R Bayona J Blöndal K Caminero JA Cegielski JP Danilovits M Espinal MA Hollo V Jaramillo E Leimane V Mitnick CD Mukherjee JS Nunn P Pasechnikov A Tupasi T Wells C Raviglione MC Multidrug-resistant tuberculosis management in resource-limited settings Emerg Infect Dis 2006 12 1389 1397 17073088 10.3201/eid1209.051618 PMC3294733

[B86] Chaisson RE Clermont HC Holt EA Cantave M Johnson MP Atkinson J Davis H Boulos R Quinn TC Halsey NA Six-month supervised intermittent tuberculosis therapy in Haitian patients with and without HIV infection Am J Respir Crit Care Med 1996 154 1034 1038 8887603 10.1164/ajrccm.154.4.8887603

[B87] Desvarieux M Hyppolite PR Johnson WD Jr Pape JW A novel approach to directly observed therapy for tuberculosis in an HIV-endemic area Am J Public Health 2001 91 138 141 10.2105/AJPH.91.10.1547 11189809 PMC1446511

[B88] Johnson JL Okwera A Vjecha MJ Byekwaso F Nakibali J Nyole S Milberg J Aisu T Whalen CC Mugerwa RD Ellner JJ Risk factors for relapse in human immunodeficiency virus type 1 infected adults with pulmonary tuberculosis Int J Tuberc Lung Dis 1997 1 446 453 9441100

[B89] Sonnenberg P Murray J Glynn JR Shearer S Kambashi B Godfrey-Faussett P HIV-1 and recurrence, relapse, and reinfection of tuberculosis after cure: a cohort study in South African mineworkers Lancet 2001 358 1687 1693 10.1016/S0140-6736(01)06712-5 11728545

[B90] Pulido F Peña JM Rubio R Moreno S González J Guijarro C Costa JR Vázquez JJ Relapse of tuberculosis after treatment in human immunodeficiency virus-infected patients Arch Intern Med 1997 157 227 231 10.1001/archinte.157.2.227 9009982

[B91] Cummings KC Mohle-Boetani J Royce SE Chin DP Movement of tuberculosis patients and the failure to complete antituberculosis treatment Am J Respir Crit Care Med 1998 157 1249 1252 9563747 10.1164/ajrccm.157.4.9708058

[B92] Roos BR van Cleeff MRA Githui WA Kivihya-Ndugga L Odhiambo JA Kibuga DK Klatser PR Cost-effectiveness of the polymerase chain reaction versus smear examination for the diagnosis of tuberculosis in Kenya: a theoretical model Int J Tuber Lung Dis 1997 2 235 241 9526197

[B93] Trunz BB Fine P Dye C Effect of BCG vaccination on childhood tuberculous meningitis and miliary tuberculosis worldwide: a meta-analysis and assessment of cost-effectiveness Lancet 2006 367 1173 1180 10.1016/S0140-6736(06)68507-3 16616560

